# Cross-Species Metabolic Profiling of Floral Specialized Metabolism Facilitates Understanding of Evolutional Aspects of Metabolism Among Brassicaceae Species

**DOI:** 10.3389/fpls.2021.640141

**Published:** 2021-03-31

**Authors:** Yuting Liu, Mutsumi Watanabe, Sayuri Yasukawa, Yuriko Kawamura, Chaiwat Aneklaphakij, Alisdair R. Fernie, Takayuki Tohge

**Affiliations:** ^1^Graduate School of Biological Science, Nara Institute of Science and Technology (NAIST), Ikoma, Japan; ^2^Department of Pharmacognosy, Faculty of Pharmacy, Mahidol University, Bangkok, Thailand; ^3^Max-Planck-Institute of Molecular Plant Physiology, Potsdam-Golm, Germany

**Keywords:** floral specialized metabolite, plant metabolomics, cross-species comparison, chemodiversity, Brassicaceae, compatible and incompatible species, flavonoids

## Abstract

Plants produce a variety of floral specialized (secondary) metabolites with roles in several physiological functions, including light-protection, attraction of pollinators, and protection against herbivores. Pigments and volatiles synthesized in the petal have been focused on and characterized as major chemical factors influencing pollination. Recent advances in plant metabolomics have revealed that the major floral specialized metabolites found in land plant species are hydroxycinnamates, phenolamides, and flavonoids albeit these are present in various quantities and encompass diverse chemical structures in different species. Here, we analyzed numerous floral specialized metabolites in 20 different Brassicaceae genotypes encompassing both different species and in the case of crop species different cultivars including self-compatible (SC) and self-incompatible (SI) species by liquid chromatography-mass spectrometry (LC-MS). Of the 228 metabolites detected in flowers among 20 Brassicaceae species, 15 metabolite peaks including one phenylacyl-flavonoids and five phenolamides were detected and annotated as key metabolites to distinguish SC and SI plant species, respectively. Our results provide a family-wide metabolic framework and delineate signatures for compatible and incompatible genotypes thereby providing insight into evolutionary aspects of floral metabolism in Brassicaceae species.

## Introduction

Self-compatibility and self-incompatibility are common reproductive systems in flowering plant species, with both modes having advantages for species reproduction and selective breeding. Self-compatible (SC) species can integrate reproductive assurance with low-cost effects (inbreeding depression; Lahiani et al., 2015) and rapidly occupy a suitable growth habitat, but lack adaptability to sudden environmental changes. By contrast, self-incompatible (SI) species have increased individual genetic heterozygosity, which accelerates species differentiation to adapt to environmental changes (Charlesworth et al., 2005); however, as a consequence they pay higher costs, such as the need to attract pollinators. Given the benefits of outcrossing, SC species can, with the help of pollinators, disperse more seeds (Kobayashi et al., 2012). Consequently, both SC and SI species have developed techniques to attract pollinators, but SI species have a higher demand for pollinators and therefore invest more in this process. For SI plants, flowers are the most important reproductive organ and bear the considerable task of attracting pollinators. To do so they rely on signals such as attractive floral pigmentation patterns, large floral size, strong floral scent, or rich nectar-honey rewards. In *Raphanus raphanistrum* and *Antirrhinum* spp., yellow-flowered individuals attract more attention from bees, thus obtaining more visits than white-flowered individuals (Stanton et al., 1986; Jones and Reithel, 2001). In monkeyflowers (*Mimulus* spp.), the presence or absence of yellow carotenoids, which are regulated by *YUP* genes, significantly shifts the preference of bees and hummingbirds (Bradshaw and Schemske, 2003). In addition to overall flower color, colorful pigmentation patterns such as spots, stripes, and central deepening or brightening of hue can serve as a nectar guide to promote pollination success (Davies et al., 2012). In *Mimulus lewisii*, loss of “light areas,” which are formed by the flux from anthocyanin pigments to colorless flavonols, results in a significantly decreased rate of bumblebee visitation (Yuan et al., 2016). In addition to the pigmentation patterns that can be recognized by human eyes, UV-absorbing areas ranging from UV-C to UV-A (~300–400 nm) on flowers serve as a UV nectar guide, which can be sensed by some insect species (Kevan, 1976). Flavonoids usually play a role in this process, promoting visitation from pollinators such as bees or flies. Some accessions of *Brassica rapa* that present a UV “bulls-eye” nectar guide pattern on their corolla gain greater preference from pollinators. The UV-absorbing compound isorhamnetin 3,7-*O*-di-glucoside underlies UV patterning in these plants (Sasaki and Takahashi, 2002; Brock et al., 2016). In *Rudbeckia hirta*, 19 flavonols from the basal UV-absorbing area and apical UV-reflecting area on petals have been characterized, most of which accumulate in the basal area. Moreover, flavonol 7-*O*-glucosides showing yellow fluorescence under UV light are found to exclusively accumulate in the basal part and may contribute to promoting the sight-sensibility of pollinators (Schlangen et al., 2009).

To adapt to demand as a reproductive organ, specialized (secondary) metabolites in flowers are also needed to ensure the success of the fertilization process, such as protecting pollen from UV-irradiation and herbivores. Pollen from flowers of SI plant species may require a long trip to drop on suitable stigma, so stronger protection against UV-B stress, which reduces viable pollen production (Demchik and Day, 1996), inhibits pollen germination and tube growth (Feng et al., 2000) and makes pollen shrivel (Koti et al., 2005), is needed. UV-absorbing flavonols or other compounds with phenolic acid moieties, as well as some phenolamides, can play an important antioxidant role in response to UV irradiation. Levels of flavonols such as quercetin and kaempferol glucoside derivatives have frequently been reported to significantly increase in *Brassica napus*, *Trifolium repens*, *Malus domestica*, and *Arabidopsis thaliana* when plants are exposed to UV radiation (Olsson et al., 1998; Hofmann et al., 2000; Solovchenko and Schmitz-Eiberger, 2003; Götz et al., 2010). Phenylacylated-flavonols (saiginols) in floral tissue of accessions of *Arabidopsis* found at low-latitude and high-altitude have been shown to confer greater UV light tolerance (Tohge et al., 2016). Moreover, levels of hydroxycinnamic acids, such as caffeic acid, ferulic acid, sinapoyl-malate, and sinapoyl-*O*-glucoside, also increase greatly in response to UV treatment in tomato, red leaf lettuce, *A. thaliana*, and *B. rapa* (Luthria et al., 2006; García-Macías et al., 2007; Meißner et al., 2008; Li et al., 2010; Brock et al., 2016). Additionally, phenolamides, a large proportion of which are phenolic acids, emerge as the major metabolites in anther and pollen grains, and also function to strengthen abiotic resistance and affect fertility (Bassard et al., 2010). To date, metabolic profiling of floral organisms has been explored to elucidate the function of flower-specific specialized metabolites. For example, spatial metabolite expression in the flower has been described for *Fragaria*×*ananassa*, *Crocus sativus*, and *Rumex algeriensis* (Hanhineva et al., 2008; Moraga et al., 2009; Ammar et al., 2020). However, the metabolic profiling of multiple closely related species still needs to be focused on in order to further elucidate evolutionary aspects of floral metabolism. Specific classes of specialized metabolites in flowers, such as flavonoids or phenolamides, have already been discussed. Furthermore, specialized metabolites, such as pollen-specific compound *N'*,*N''*-di-(hydroxyferuloyl)-*N'''*-sinapoyl spermidine and flavonol-3-*O*-diglucosides (kaempferol/quercetin-3-*O*-(2''-*O*-glucosyl)glucosides), have also been characterized (Fellenberg et al., 2009; Yonekura-Sakakibara et al., 2014), therefore, their roles in structural and functional diversification are worthy of discussion.

Brassicaceae, which contains many agriculturally important SC and SI species, such as *A. thaliana* and many of its relatives, have SC reproductive systems (Roy et al., 2010). On the other hand, many species in the *Brassica* genus have an SI reproductive system. For instance, *B. rapa* (Br) and *Raphanus sativus* (Rs) have an SI system, yet the amphidiploid species *Brassica juncea* (Bj) and *B. napus* (Bn) have incomplete SC systems (Kobayashi et al., 2012). So far, the SI model system in Brassicaceae has been well-characterized at the molecular level. In *B. napus*, the SI reaction is initiated by allele-specific recognition of the pollen-coat protein SCR/SP11 by *S*-locus receptor kinase (SRK). The activation of SRK causes activation of E3 ligase ARC1, which degrades compatibility factors such as Exo70A1, GLO1, and phospholipase D, which results in pollen rejection (Charlesworth et al., 2005; Kitashiba and Nasrallah, 2014; Scandola and Samuel, 2019). However, to date, only a few studies have systematically compared the differences in metabolite composition between compatible and incompatible species. In this study, we employed an LC-MS-based metabolomic approach to describe the metabolic framework of specialized floral metabolism as a whole from both compatible and incompatible species in Brassicaceae. This has provided insight into the cross-species metabolites featured among different clades, as well as the role of specific metabolites in response to the reproductive recognition system.

## Materials and Methods

### Plant Materials

Plant genotypes including *A. thaliana* (accessions Col-0, C24, and *tt4* mutant), *Arabidopsis shokei* (As), *Arabidopsis lyrate* (Al), *Crucihimalaya lasiocarpa* (Cl), *Olimarabidopsis pimila*, (Op) *Lepidium sativum* (Ls), *Thellungiella salsuginea* (Ts), *Thlaspi arvense* (Ta), *Capsella rubella* (Cr), *Nasturtium officinale* (No), *Cardamine hirsute* (Ch), *R. sativus* (Rs), *Brassica oleracea* var. *alboglabra* (Boa), *Brassica oleracea* var. italic (Boi), *B. napus* (Bn), *B. rapa* (Br), *Sinapis alba* (Sa), *Diplotaxis muralis* (Dm), *Eruca sativa* (Es), and *B. juncea* (Bj) were obtained from the Arabidopsis Biological Resource Center (ABRC),^1^ and the seed companies (Takii, Japan; Sakata-no-Tane, Japan and Marche, Japan). Plants were grown in the greenhouse at 22°C for long day condition (16 h light/8 h dark). Those plants were grown in a mixture of red jade soil-vermiculite-nutrient soil (1:3:7). More than two whole flowers (containing stamen, petal, pistil, and anther) were collected in individual three biological replicates from two to three individual plants at the day of full-opened, and frozen immediately in liquid nitrogen. Samples were ground by Mixer Mill TissueLyser II (Qiagen, Hilden, Germany). The frozen plant powder was stored at −80°C until use.

### Phylogenetic Tree Construction

The internal transcribed spacer (ITS) sequences from Brassicaceae species were retrieved from the NCBI database. Accession numbers are displayed in Table 1. The sequences were aligned by the MUSCLE algorithm (Edgar, 2004). The positions comprising more than 70% unrecognized characters were discarded. A phylogenetic tree was constructed by molecular evolutionary genetics analysis version 10.0 (MEGA X; Kumar et al., 2018) by the maximum likelihood (ML) method. Model selection for ML analysis was performed by using the model selection tool as supplemented in MEGA X. The model test result showed that the general time reversible model was the most suitable for analysis. The initial tree for the heuristic search was obtained automatically by applying Neighbor-Join and BioNJ algorithms to a matrix of pairwise distances estimated using the Maximum Composite Likelihood (MCL) approach, and then selecting the topology with superior log likelihood value. A discrete Gamma distribution was used to model evolutionary rate differences among sites (five categories, +G, and parameter). The tree confidence was inferred by the bootstrap method containing 1,000 replicates (Felsenstein, 1985). *Cleome serrulata* (accession number DQ455804.1) was selected as an outgroup.

**Table 1:** Twenty Brassicaceae genotypes analyzed in this study.

ID	Genotypes (species/cultivars)	Reproductive system	GenBank ID^**^	Reference
At	*thaliana*	SC^*^	MG886682.1	Zhang et al., 2019
As	*shokei*	SC	N/A	Indriolo et al., 2012
Al	*lyrata*	SI	DQ528878.1	Indriolo et al., 2012
Op	*O. pumila*	SC	DQ310528.1	Roy et al., 2010
Cl	*C. lasiocarpa*	SC	AF137556.1	Roy et al., 2010
Ls	*L. sativum*	SC	MN257764.1	Wadhwa et al., 2012
Ch	*C. hirsura*	SC	DQ268383.1	Hay and Tsiantis, 2006
No	*N. officinale*	SC	AY254531.1	Pink, 1993
Cr	*C. rubella*	SC	AY662286.1	Fujikura et al., 2018
Ta	*T. arvense*	SC	KM892656.1	Best and McIntyre, 1975
Ts	*T. salsuginea*	SC	DQ165371.1	Wang et al., 2015
Rs	*R. sativus*	SI	GQ268079.1	Roy et al., 2010
Bj	*juncea*	Incomplete SC	MG923970.1	Kobayashi et al., 2012
Boa	*B. oleracea* var. alboglabra	SC	GQ891870.1	Okuda et al., 1997
Boi	*B. oleracea* var. italica	SI	KX709353.1	Anstey, 1954
Bn	*B. napus*	Incomplete SC	MG923974.1	Kobayashi et al., 2012
Br	*B. rapa*	SI	MG923989.1	Roy et al., 2010
Sa	*S. alba*	SC	AF128106.1	Fan et al., 2007
Dm	*D. muralis*	SC	DQ983972.1	Kokichi and Noboru, 1976
Es	*E. sativa*	SI	AY254536.1	Wang et al., 2007

∗ SC indicates self-compatible; SI indicates self-incompatible; Incomplete SC indicates incomplete self-compatible.

∗∗ N/A indicates not available in NCBI database.

### Metabolite Extraction

Metabolite extraction was conducted as previously described (Tohge and Fernie, 2010). About 15 mg of frozen sample was weighed. Extraction buffer i.e., 80% methanol (5 μg/ml isovitexin as an internal standard) was added at a ratio of 1 mg F.W. sample powder: 10 μl extraction buffer. The mixture was vortexed to mix thoroughly and centrifuged at 14,000 rpm at 4°C for 10 min, then supernatant was transferred into new 1.5 ml tube and centrifuged once more, the final obtained supernatant was transferred into vials for liquid chromatography-mass spectrometry (LC-MS) and high performance liquid chromatography-photodiode array detector (HPLC-PDA).

### LC-MS Analysis

The flower metabolite extracts of 20 genotypes *A. thaliana* C24, *A. shokei*, *Arabidopsis lyrata*, *C. lasiocarpa*, *O. pimila*, *L. sativum*, *T. salsuginea*, *T. arvense*, *C. rubella*, *N. officinale*, *C. hirsute*, *R. sativus*, *B. oleracea* var. alboglabra, *B. oleracea* var. italic, *B. napus*, *B. rapa*, *S. alba*, *D. muralis*, *E. sativa*, and *B. juncea* were used for LC-MS analysis. LC-MS was carried out as described previously (Calumpang et al., 2020). Chromatographic separations were conducted on Nanoflow-HPLC “Paradigm MS4 system” (Michrom BioResources, Inc., Auburn, CA, United States), equipped with a Luna C18 column (150 by 2.00 mm i.d. 3 micron particle size, Phenomenex, Torrance, CA, United States). The mobile phase consisted of Solvent A (0.1% formic acid in water) and B (0.1% formic acid in acetonitrile). For each injection, 10 μl sample was loaded, and following gradient was applied at a flow rate of 200 μl min^−1^: 0–1 min, from 100% A to 93%; 1–8 min, to 80% A; 8–17 min, to 60% A; 17–21 min, to 15% A; 21–25 min, to 0% A; 25–28 min, column wash; 28–31 min, to 100% A for equilibration of the column. Compounds were detected from *m/z* 200–1,500 by MS TSQ Vantage (Thermo Fisher Scientific, San Jose, CA, United States) using full scan mode covering both positive and negative ion detection. The transfer capillary temperature was set to 350°C and the spray voltage was fixed at 3.00 kV. The chromatograms were analyzed by Xcalibur software version 4.1.31.9 (Thermo Fisher Scientific, San Jose, CA, United States), the *m/z* value, retention time, and detection mode information of 228 characteristic peaks from all 20 genotypes were extracted and listed for peak picking process. Peak picking was conducted by using the process program in Xcalibur software to deal with the raw files of 20 genotypes with the parameter of retention time tolerance window (20 s), base window 100, area noise factor 5.0, peak noise factor 10, mass tolerance 0.5 *m/z*, and “nearest RT.” A data matrix of areas of extracted ion chromatogram (EIC) was exported into Microsoft Excel and used for statistical analysis. Peak annotation of major flavonols, phenolic acid derivatives, phenolamides, and glucosinolates (GSLs) were conducted *via* by combined approach of literature survey, databases, co-elution of reference plant-extracts of *Arabidopsis* (Col-0, C24, and *tt4* mutant) and profiling of specific in-source fragments detected in positive ion detection as well as retention time referring to the metabolite information from the literature (Hanhineva et al., 2008; Fellenberg et al., 2009; Tohge et al., 2016) and databases including m/zCloud,^2^ KNApSAcK (Afendi et al., 2012),^3^ and PubChem.^4^ Consequently, 82 peaks and 16 peaks were annotated or classified to compound class out of 228 characteristic peaks (Supplementary Table S1).

### HPLC-PDA Analysis

The flower metabolite extraction of *R. sativus* and *A. thaliana* accession C24, Col-0 as well as *tt4* mutant were used for HPLC analysis. The HPLC analysis was conducted on Waters alliance HPLC system (Waters, Milford, MA, United States) controlled by Empower™ 25 (Waters, United States) software. Chromatographic separation was achieved on Waters 2695 separations module equipped with a 00F-451-B0 column (150 by 2.0 mm i.d. 3 micron particle size, Phenomenex, Torrance, CA, United States). The Waters 2996 photodiode array detector was employed for UV/VIS-detecting at 200~550 wavelength range. The mobile phase consisted of Solvent A (0.1% formic acid in water) and B (0.1% formic acid in acetonitrile). The sample injection volume was 10 μl each time. The flow-rate was set to 200 μl min^−1^. Following elution program was performed for separation: 0–2 min, from 0% B to 10%; 2–15 min, to 25% B; 15–27 min, to 55% B; 27–30 min, to 100% B; 35–35.01 min, to 0% B; 35. 01–40 min, column wash.

### Statistical Analysis

The peak area value of 228 characteristic peaks in each genotype was normalized by multiplication with the ratio of the average peak area of the internal standard to the peak area of the internal standard. The average of three replicates of normalized peak area of 82 annotated metabolites was then calculated and used for creating percentage stacked column shown in Figure 2 by using Microsoft excel 2016 to represent the proportion of each annotated compound in different groups. The average of three replicates of normalized peak area of 228 characteristic peaks was scaled by log_2_ (mean/average_mean) and used for heatmap analysis. Heatmap visualization of metabolite data was performed by MeV software version 4.9.0 (Dana Farber Cancer Institute, Boston, MA, United States).^5^ Metabolites and genotypes in heatmap were clustered using hierarchical clustering method (HCL). The normalized peak area value with three replicates of 228 characteristic peaks was used for the K-means test, principal component analysis (PCA) and partial least squares-discriminant analysis (PLS-DA), which were conducted by MetaboAnalyst 4.0 (Xia and Wishart, 2011).^6^ The missing value was replaced by 1/5 of the min positive value for each variable. The peak area value of metabolites with high variations (RSDs > 20% in QC samples) were excluded and data was normalized by following parameters: none row-wise normalization, logarithmic 10 transformation, and none-data scaling. The significant component of PLS-DA was calculated using 10-fold cross-validation, and the predictive ability was evaluated by Q^2^ value. The metabolites with variable importance in projection (VIP) scores greater than 1.7 were considered as significant feature metabolites. The absorption wavelength value (250~520 nm) of HPLC data was extracted by Empower™ 25 (Waters, United States) software, and scaled by setting the lowest value and highest value as 0 and 1. The cluster analysis of HPLC spectrum was conducted by K-means in MeV software.^7^ The number of clusters was decided by formula $\sqrt{\frac{n}{2}}$ (n represent the number of input data; Joseph et al., 2010).

## Results

### Phylogenetic Relationship and Floral Phenotype of 20 Brassicaceae Genotypes

**Figure 1:**
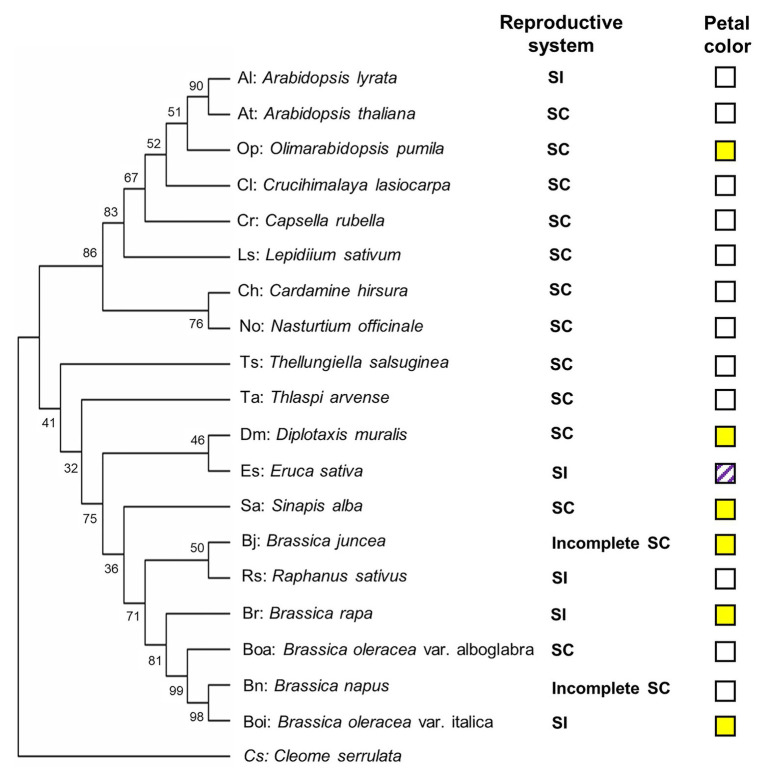
Phylogenetic diagram of the relationships among selected Brassicaceae genotypes. The phylogenetic tree was constructed by MEGA X (Kumar et al., 2018) using the sequence of the internal transcribed spacer (ITS) gene of each genotype. *Cleome serrulata* was set as outgroup. The maximum likelihood (ML) method was used with the following parameters: General time reversible model, complete deletion and bootstrap (1,000 replicates). Values on the branches indicate bootstrap support in percentages. SC indicates self-compatible. SI indicates self-incompatible, incomplete SC indicates incomplete self-compatible. White square indicates white flower color, yellow square indicates yellow flower color, and white squares with purple stripes indicate white flower with purple stripes.

In this study, 20 genotypes encompassing species and cultivars of the different subfamilies of the Brassicaceae were selected, including 12 SC species (two cultivars for *B. oleraceae*), two incomplete SC species, and five SI species (Table 1). To construct phylogenic relationship among selected Brassicaceae plant species, the phylogenetic tree was constructed by the sequence of the ITS gene of each species (Figure 1). Furthermore, visible color of flower petal was checked for consideration of indication to plant speciation, floral pigmentation, and pollinators’ recognition. Fourteen of twenty species had white flowers including *A. thaliana* (At), *A. shokei* (As), *A. lyrata* (Al), *C. lasiocarpa* (Cl), *L. sativum* (Ls), *C. hirsuta* (Ch), *N. officinale* (No), *C. rubella* (Cr), *T. arvense* (Ta), *T. salsuginea* (Ts), *R. sativus* (Rs), *B. oleracea* var. alboglabra (Boa), *B. napus* (Bn), and *E. sativa* (Es). Among these species, *E. sativa* had special purple stripes. Six species had bright yellow flowers including *Ophiorrhiza pumila* (Op), *B. juncea* (Bj), *B. oleracea* var. italica (Boi), *B. rapa* (Br), *S. alba* (Sa), and *D. muralis* (Dm) (Figure 1). Within six SI plant species, four Brassicaceae plants except *A. lyrata* (Al) and *R. sativus* (Rs) were species producing pigmented petals.

### Flower Metabolite Profiling of the 20 Brassicaceae Genotypes

**Figure 2:**
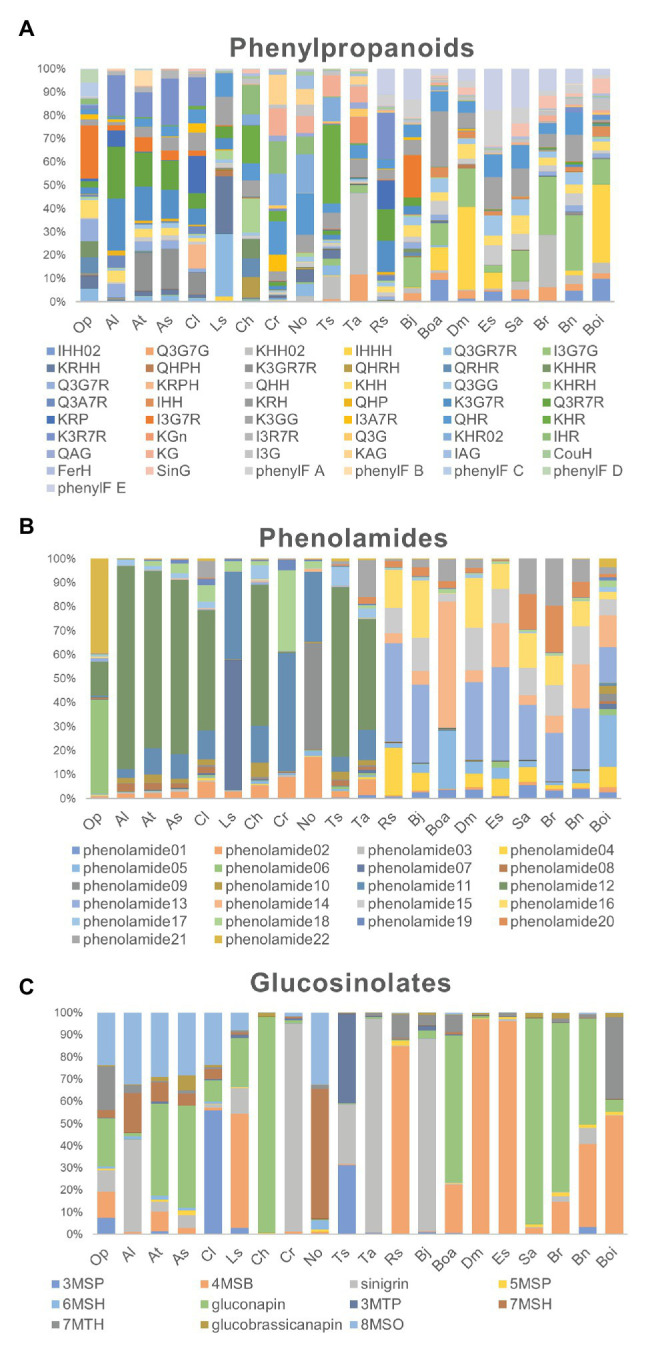
Relative proportion of metabolites in each genotype. **(A)** The proportion of 49 phenylpropanoid compounds in each genotype; **(B)** The proportion of 22 phenolamides in each genotype; **(C)** The proportion of 11 glucosinolate compounds in each genotype.

**Figure 3:**
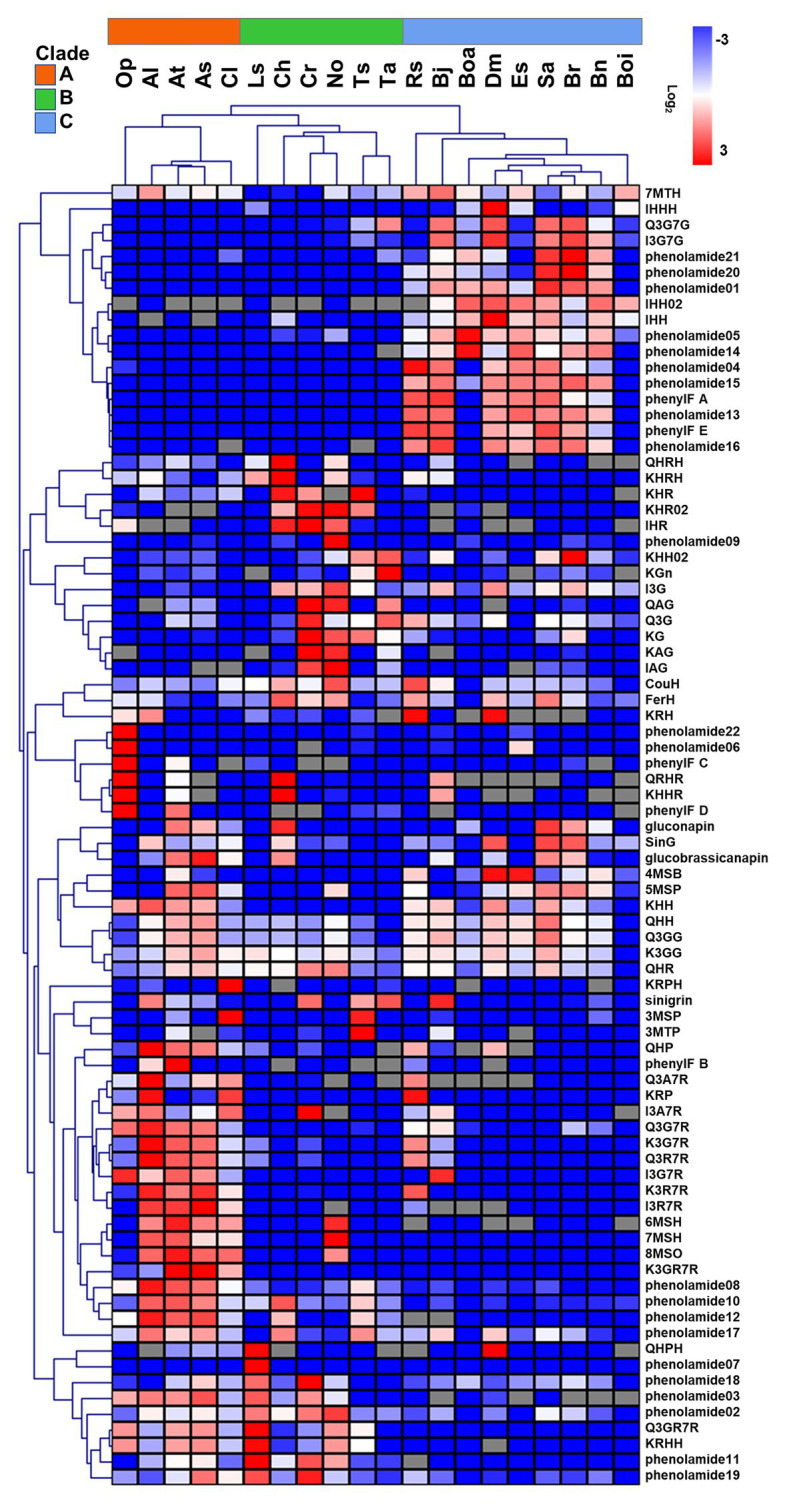
Relative abundance of 82 annotated metabolites in floral organ of 20 genotypes. Color indicates the level of log_2_ (mean/mean_average). Gray grids indicate no detection. Metabolites and genotypes in heatmap were clustered using hierarchical clustering method (HCL).

To evaluate the metabolic variance across these genotypes a non-targeted metabolite profiling of entire flowers was conducted based on LC-MS. In total, 228 peaks were detected, including 82 peaks annotated as 46 flavonoids, three hydroxycinnamate derivatives, 22 phenolamides, and 11 GSLs by co-elution of *Arabidopsis* Col-0 flower extracts and combination approach with literature-based peak annotation (Figure 2; Tohge et al., 2016; de Souza et al., 2017). The relative abundance level of each peak was evaluated and presented in Supplementary Figure S1 and Supplementary Table S1. Figure 3 shows a heatmap presenting relative abundance of 82 annotated specialized metabolites. According to the clustering result of annotated peaks using hieratical clustering analysis (HCA), plant species were clustered into three clades (Table 2), which were mostly similar to their phylogenetic relationship. In the result of classification, plant species of clades A and B, with the exception of *A. lyrata*, were all self-compatible species. In clade C with the exception of *B. oleracea* var. alboglabra, *S. alba* and *D. muralis*, which were self-compatible and *B. juncea*, *B. napus* were incompletely self-compatible species, all other genotypes were self-incompatible.

**Table 2:** Clustering result using K-means.

Cluster	Species
A	At, As, Al, Cl, Op
B	Ls, Ch, No, Cr, Ta, Ts
C	Rs, Bj, Boa, Boi, Bn, Br, Sa, Dm, Es

### Diversity in Floral UV-Absorbing Phenylpropanoids Across Genotypes

Differences in the abundance of phenolic acid derivatives and flavonols were analyzed among the 20 genotypes in order to evaluate evolutionary changes in the productivity of UV-absorbing compounds. In this analysis, three hydroxycinnamate-glucosides were annotated. Sinapoyl-*O*-glucoside, a major compound in UV-nectar area in *B. rapa*, largely accumulated in the compatible species *A. thaliana*, *A. shokei*, *C. lasiocarpa*, *C. hirsuta*, *S. alba*, and *D. muralis* as well as the incompatible species *B. rapa*. The other two common compounds coumaroyl-hexose and feruloyl-hexose, were ubiquitously present across the genotypes studied, however, they were present at considerably lower abundance in clade A species. Asides from phenolic acid compounds, 46 flavonols have been detected, mostly being the mono-, di-, and tri- hexose or pentose substituents of quercetin, kaempferol, and isorhamnetin aglycones (Figure 3). The ubiquitous “UV-nectar” compound namely isorhamnetin 3,7-*O*-glucoside was found to mainly in clade C species (with the exception of *R. sativus*). The *Arabidopsis* (C24) floral phenylacyl-flavonol glycoside kaempferol-3-*O*-(2''-*O*-rhamnosyl-6''-*O*-sinapoyl)glucoside-7-*O*-rhamnoside, which was supposed to have stronger UV-defense ability because of its additional phenyl moiety specifically accumulate in *A. thaliana* (C24) and *A. lyrata*. Flavonols quercetin-3-*O*-(6''-*O*-glucosyl) glucoside and kaempferol-3-*O*-(6''-*O*-glucosyl) glucoside, which have been reported being pollen specific compound in *A. thaliana* (C24), were found to accumulate in all species except *B. oleracea* var. italica, while kaempferol-3-*O*-(rhamnosyl)glucoside-7-*O*-rhamnoside, which was detected in *A. thaliana*, only accumulated to high levels in *A. thaliana*, *A. shokei*, *C. lasiocarpa*, *L. sativum*, and *B. oleracea* var. alboglabra.

According to the flavonol composition ratio in floral organs in each species, the flavonols which contributed the greatest proportion to the total flavonol level and the changes in these rates along with evolutionary relationships were investigated. In clade A genotypes, kaempferol derivatives such as kaempferol-3-*O*-rhamnoside-7-*O*-rhamnoside, kaempferol-3-*O*-glucoside-7-*O*-rhamnoside, and kaempferol-3-*O*-(2''-*O*-rhamnosyl)-glucoside-7-*O*-rhamnoside constituted a large proportion of flavonols. For clade B, the major flavonols were still kaempferol however with different sugar moiety substitutions such as kaempferol-hexose-rhamnoside and kaempferol-hexose. Although the proportion of the isorhamnetin-hexose, isorhamnetin-hexose-rhamnoside, and isorhamnetin-3,7-*O*-di-glucoside, is low in clade B genotypes, the compounds are present whereas they are not in clade A genotypes, and expanded to represent a large proportion clade C along genotypes. The latter expansion was coupled to an obvious decrease in the proportion of quercetin and kaempferol-derivatives. In addition, two phenylacylated-flavonols were specifically detected in clade C genotypes where they constitute a large proportion of the total flavonols.

### Genotype-Specific Changes in the Type and Abundance of Floral Phenolamides

Comparison of phenolamide content was next conducted in order to investigate the evolutionary changes adapt to different environments and the divergence of reproductive system type because of their important roles in abiotic stress defense and fertility, respectively. In this study, 22 phenolamide compounds were annotated and their variance was studied in the same manner as for the flavonols described above. All genotypes in clade A and *Cardamine hirsta*, *T. salsuginea* in clade B displayed a high proportion of the well-known pollen coat constitution compound *N'*,*N''*-bis-(5-hydroxyferuloyl)-*N'''*-sinapoyl spermidine that was previously found from *A. thaliana* (Fellenberg et al., 2008). By contrast, the proportion of *N'*,*N''*-bis-feruloyl-*N'''*-(5-hydroxyferuloyl) spermidine was higher in clade B compared to clade A, being absent in clade C and *O. pumila* whose major constituent was *N'*, *N''*-di-coumaroyl spermidine. In clade C species, the proportion of *N'*,*N''*-bis-coumaroyl spermidine was greatly enhanced. In addition, the (5-hydroxyferuloyl) polyamine derivatives and caffeoyl phenolamide derivatives were specifically observed in clade C genotypes where they accounted for a large proportion of total phenolamides.

### Aliphatic Glucosinolates Are the Major Type of Glucosinolate in Floral Organs

As a unique group of specialized metabolites in Brassicaceae, GSLs were focused because of their unique and diverse roles in biotic stress defense and interaction with insects. In this study, 11 GSLs were annotated among the 20 genotypes indicating the major type to be chain-elongated aliphatic GSLs derived from methionine. In general, four glucosinolates sinigrin (2-propenylglucosinolate), gluconapin (3-butylglucosinolate), 8-methylsulfinyloctylglucosinolate (8-MSO), and 4-methylsulfinylbutylglucosinolate (4-MSB) constitute the majority of glucosinolates, however, some genotype specific differences were observed. Sinigrin and gluconapin showed relatively universal existence cross clade, but sinigrin was present at considerably higher proportions in *B. juncea*, *T. arvense*, and *C. rubella*, while gluconapin was the major glucosinolate in *A. shokei*, *C. hirsuta*, *B. rapa*, and *S. alba*. By contrast, 4-MSB was considerable in clade C genotypes especially in *E. sativa*, *D. muralis*, *R. sativus*, *B. oleracea* var. italica and part of clade A or B genotypes like *A. thaliana* and *L. sativum*. While 8-MSO occupied certain proportion mostly in *A. thaliana* relatives.

### PCA and PLS-DA Analysis to Identify Metabolite Features Cross-Clades

**Figure 4:**
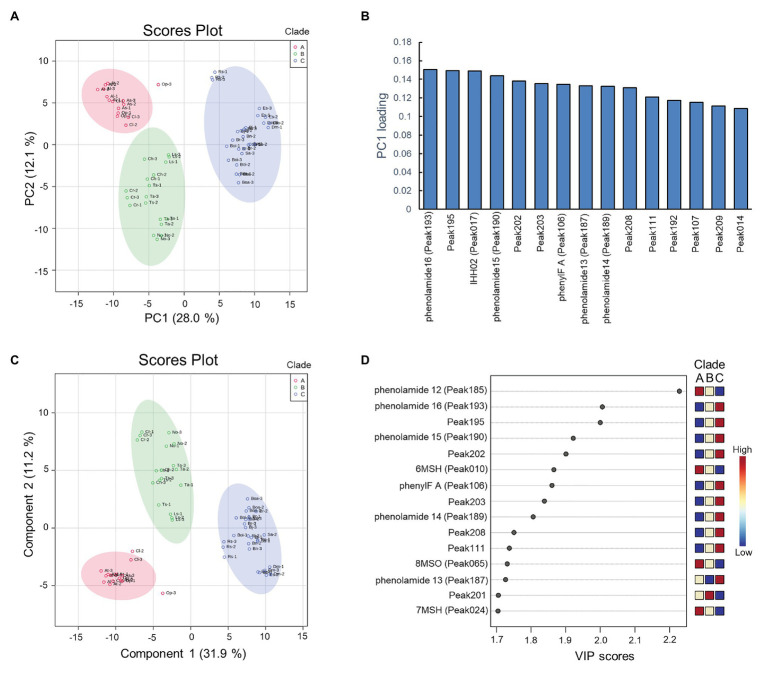
Results of principal component analysis (PCA) and partial least squares-discriminant analysis (PLS-DA) of the metabolite data. **(A)** PCA scores plot between the principal component 1 (PC1) and PC2. The explained variances are shown in brackets, colors of circle indicate three replications of metabolite data of species; **(B)** PCA loading plots between the PC1 and PC2; **(C)** PLS-DA scores plot between the PC1 and PC2. The explained variances are shown in brackets; **(D)** Important features identified by variable importance in projection (VIP) scores. The colored boxes on the right indicate the relative concentrations of the corresponding metabolite in each clade.

To assess specific floral metabolites which contribute to the discrimination of clades, the peak area value of the 228 characteristic peaks were subjected to PCA (Figure 4A). The *R*^2^ value (which is defined as the proportions of variances explained by model was used to describe the goodness of fit) of 0.401 [0.28 value of variance was captured by principal component 1 (PC1)] was generated from the PCA model using two components, unraveling the existence of differences among these three clades. To investigate the contributors to the principal components, loading plots were visualized to reveal the role of metabolites in separating clades (Figure 4B). The metabolic loadings with highest values were caffeoyl-phenolamide derivative in PC1, indicating its strong impact on separation.

To optimize the separation among three clades, PLS-DA was applied. The predictive ability of PLS-DA model was estimated by *Q*^2^ value, which was calculated *via* cross-validation (CV). As a result of 10-fold CV, the model was built with a *Q*^2^ value of 0.937, indicating this model having well predictive ability since *Q*^2^ > 0.9. As observed in the score plots of PLS-DA, species were clearly separated into three groups with 31.9% of variance was captured by PC1 (Figure 4). The VIP, a weighted sum of squares of the PLS loadings, was calculated in PLS-DA model, and metabolites with VIP > 1.7 were considered to be significantly distinguished. As a result, fifteen out of 228 characteristic peaks were detected as significant features (Figure 4). The relative levels of these significant metabolites were investigated in each clade and found to show specific pattern of presence/absence across clades. The phenylacylated flavonoid, caffeoyl phenolamide derivatives, and (5-hydroxyferuloyl) polyamine derivative displayed high accumulation in the clade C group. By contrast, the *N'*,*N''*-bis-(5-hydroxyferuloyl)-*N'''*-sinapoyl spermidine, 6-methylsufinylhexyl GSL, 7-methylsufinylheptyl GSL, and 8-methylsulfinyloctyl GSL displayed high accumulation in the clade A group, while species in clade B group almost contained medium levels of these compounds.

### HPLC Verification of Phenylacylated-Flavonoid Derivatives in *Raphanus sativus* From Clade C

From PLS-DA analysis, several putative phenylacylated-flavonoid derivatives were supposed to be the significant feature to distinguish three clades. To provide further support that they are phenylacyl-moiety decorated flavonoids, *R. sativus* which accumulated the highest abundance of these compounds was selected for performing validatory HPLC-PDA analysis. The spectrums of saiginol A (Tohge et al., 2016), a group of phenylacylated-flavonoid compounds from the C24 accession of *A. thaliana* were used as positive control. Additionally, the spectrums of major flavonol-glycosides, kaempferol-3-*O*-(rhamnosyl)glucoside-7-*O*-glucoside, kaempferol-3-*O*-glucoside-7-*O*-rhamnoside and kaempferol-3-*O*-rhamnoside-7-*O*-rhamnoside, and hydroxycinnamate, sinapoyl-malate of *Arabidopsis*, were used as a negative control. Clustering by HCA of the resultant HPLC spectrum showed that the absorbance peaks at 17.57, 18.21, and 18.68 min on the chromatogram of *R. sativus* compared with saiginol A, suggesting them to be phenylacylated compounds (Supplementary Table S2). Further comparison of the absorption spectrum supported that peaks at 17.57 and 18.68 min were phenylacylated-flavonoids similar to saiginol A, while peak at 20.26 min corresponding to sinapoyl-malate shows different absorption pattern (Supplementary Figure S2).

## Discussion

With few exceptions flowers in angiosperms are responsible for producing the next generation, therefore specialized metabolites in flowers serve the purpose of attracting pollinators, playing protective roles to against biotic or abiotic stress such as UV-irradiation or herbivory, and being an important constitution allowing the maintenance of normal fertility (Tohge et al., 2018; Borghi et al., 2019; Borghi and Fernie, 2020). Successful fertilization involves certain metabolite groups, for example, phenolic acids derivatives and flavonols function as important UV-absorbing compounds, some of which are essential to maintain pollen activity (Xue et al., 2020), phenolamides play unique role for fertility (Bassard et al., 2010), and glucosinolates are involve in plant-insect interaction (Liu et al., 2020). In this study, specialized metabolites from flowers of 20 Brassicaceae genotypes were investigated in order to study the metabolite evolutionary changes concomitant to the divergence of the different clades and the underlying different reproductive recognition systems.

To attract pollinators, bright color helps the flower to be more visible. With the exception of *O. pumila* in clade A, all clade A and clade B genotypes which includes the majority of SC genotypes had white flowers, while species in clade C mostly had yellow flowers, or specific pigmentation pattern such as that exhibited by *E. sativum*, indicating that they may be more attractive to pollinators. Hence this clade C harbors predominantly SI or incomplete SC genotypes. Besides the human-visible bright colors of flowers, UV-nectar also greatly promotes pollinator visits. Indeed, sinapoyl-*O*-glucoside and isorhamnetin 3,7-*O*-di-glucoside were characterized to highly accumulate in the UV-nectar area in incompatible species *B. rapa* (Brock et al., 2016). While in our study, with the exception of *B. rapa*, sinapoyl-*O*-glucoside accumulated in self-compatible genotypes, but isorhamnetin-3,7-*O*-di-glucoside accumulated in clade C species, most of which being self-incompatible genotypes. In general, genotypes in clade C had brighter flower color and specific UV-nectar compound, in accordance with the demand of being more attractive to pollinators for most of species in clade C were self-incompatible genotypes.

As a crucial step of the fertilization process, pollen production and its activity are easily affected by UV-irradiation since enhanced UV-B levels reduce the pollen viability (Koti et al., 2005). Flavonols not only play a role in presenting UV nectar patterns on petals attracting pollinators, but also function as UV-defense compounds that protect reproductive tissues such as pollen, and be essential for fertility and pollen tube germination. Flavonols such as phenylacylated-flavonols, kaempferol, and quercetin are frequently reported to response to UV-irradiation and promote the UV tolerance (Hofmann et al., 2000; Tohge et al., 2016). As a result of the PCA and PLS-DA analysis in this study, phenylacylated-flavonoids was found as metabolite features, which discriminated the three clades, being specifically highly accumulated in clade C genotypes, indicating genotypes in clade C may have a higher demand, and therefore have evolved a higher ability, for UV-resistance.

Phenolamides accumulate in different floral organ, with some members of this compound class considered to be markers of fertility and play an important role in pollen development (Bassard et al., 2010). Moreover, phenolamides help to adapt to abiotic stress due to their antioxidant and radical scavenging ability. Additionally, spermidine derivatives are stamen-specific compounds, and their content may decrease due to the sharply decreased ratio of pollen grains to ovules, which increases the tendency of self-fertilization. *N'*, *N''*-bis-(5-hydroxyferuloyl)-*N'''*-sinapoyl spermidine is a well-known major pollen coat constituent in *A. thaliana*, and this pathway was evolved by the generation of an enzymatic cascade of six successive hydroxylations by two partially redundant cytochromes P450 genes *CYP98A8* and *CYP98A9*, which are duplications of an ancestor *CYP98A3*, showing new functionalization under Darwinian selection (Matsuno et al., 2009). In the current study, *N'*,*N''*-bis-(5-hydroxyferuloyl)-*N'''*-sinapoyl spermidine has low abundance in most genotypes of clade B and clade C, indicating possible functional differentiation of the CYP98A family in those genotypes. Di-coumaroyl and tri-coumaroyl spermidine, which are considered as markers of male fertility are abundant in anther (Werner et al., 1995), and rape bee pollen nectar (Zhang et al., 2020). In this study, the proportion of di-coumaroyl spermidine in clade C genotypes was much higher than that in clade A and B, indicating the species in clade C may attract higher bee visitation. Di-feruloyl polyamine derivatives are usually transiently detected following fertilization (Bassard et al., 2010), indicating a possible earlier fertilization of clade A and clade B genotypes than clade C genotypes.

Glucosinolates are sulfur- and nitrogen-containing specific specialized metabolites of Brassicaceae species, which have a defense activity against herbivorous insects and serve as sulfur sources under sulfur-deficiency (Aarabi et al., 2016). Glucosinolates are derived from a variety of amino acids, therefore, comprising a diverse group whose chemical diversity is defined by the side-chain modification and amino acid elongation (Kliebenstein et al., 2001). They are divided into aliphatic GSLs, indole GSLs, and aromatic GSLs according to their chemical structure. Physiological experiments in *A. thaliana* have revealed that, aliphatic GSLs function as the sulfur source during sulfur-deficiency, indole GSLs are essential for the defense and symbiosis balance toward soil fungi and nematode (Hiruma, 2019), and aromatic GSLs have specifically high accumulation in seeds involved in the inhibition of seed germination (Brown et al., 2003). Here, we demonstrate that aliphatic GSLs were the majority in floral organ. Usually, the glucosinolates are toxic to herbivores, and more recent reports have uncovered the diversity in their toxicity. For example, low-sinigrin content in plants tends to increase caterpillar attack, while glucoiberin (3-methylsulfinylpropylglucosinolate) has the opposite effect (Olsson and Jonasson, 1994). In *B. juncea, T. arvense*, *C. rubella*, and *A. lyrata*, sinigrin was the major GSL in flowers, indicating a possible lower attack rate of these species from caterpillar. A recent report shows that their corresponding diverse breakdown products volatile isothiocyanates (ITCs) can drive the host preference of crucifer-specialist moth (Liu et al., 2020). In this study, the 3-methylthiopropylglucosinolate and 4-pentenylglucosinolate, corresponding breakdown products of which are the crucifer-specialized moth-attracting volatiles ITCs iberverin and 4-pentenyl ITC, actually constituted d a very small proportion of glucosinolates in all genotypes. We suggest that this result may be the consequence of the balance between the pressure from crucifer-specialized moths and the need for toxic defenses towards other insects.

## Conclusion

In this study, we performed non-targeted metabolite profiling of 20 genotypes from different genus in Brassicaceae to explore the evolutionary aspects of floral metabolites corresponding to different reproductive recognition system. More than 228 characteristic peaks with 82 annotated specialized metabolites were detected including those from the phenolics, phenolamides, and glucosinolates class. We demonstrated that although diversity of specialized metabolites presented among species, these metabolites exhibited certain metabolic structure characteristics among different clades, which we speculate is linked to their different roles in the different fertilization processes alongside divergent evolution under biotic/abiotic stress. Through PCA and PLS-DA analysis, 15 metabolites including one phenylacylated-flavonoid and five phenolamides were indicated as significant contributors to this discrimination. These polyphenolic compounds are suggested as metabolites, which possibly have evolved during metabolic evolution conferring the physiological function as pollinator attraction and/or light protection. As such our study provides new insights into the evolutionary aspects of floral specialized metabolism and relationship to the environment.

## Data Availability Statement

The datasets presented in this study can be found in online repositories. The names of the repository/repositories and accession number(s) can be found in the article/Supplementary Material.

## Author Contributions

YL, MW, and TT conceived, designed, and conceptualized the outline of the review manuscript. MW, SY, and YK performed plant cultivation and metabolite profiling. YL and TT performed data analysis and peak annotation. YL, AF, and TT wrote the manuscript. YL, MW, CA, AF, and TT supervised and edited throughout the manuscript. All authors contributed to the article and approved the submitted version.

### Conflict of Interest

The authors declare that the research was conducted in the absence of any commercial or financial relationships that could be construed as a potential conflict of interest.

## Footnotes

^1^https://abrc.osu.edu/

^2^https://www.mzcloud.org/

^3^http://www.knapsackfamily.com/KNApSAcK_Family/

^4^https://pubchem.ncbi.nlm.nih.gov/

^5^http://www.mev.tm4.org/

^6^http://www.metaboanalyst.ca

^7^http://www.mev.tm4.org/
