# Supplementary material for: Cross-Species Metabolic Profiling of Floral Specialized Metabolism Facilitates Understanding of Evolutional Aspects of Metabolism Among Brassicaceae Species
Source: Front Plant Sci. 2021 Mar 31;12:640141. doi: 10.3389/fpls.2021.640141 (PMC8045754; doi:10.3389/fpls.2021.640141)
Supplement: Supplementary file 3 [file Image_1.pdf]

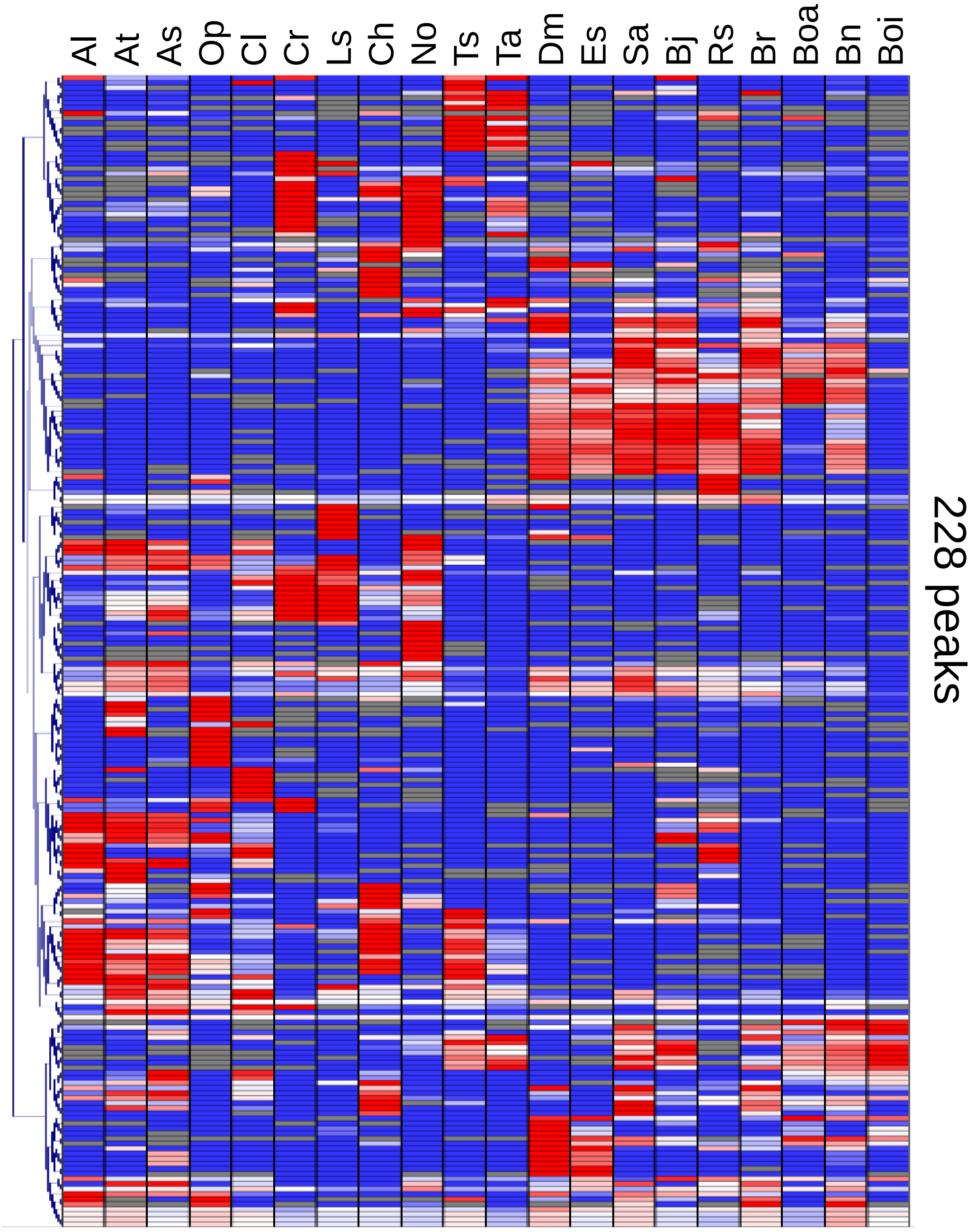

Figure S1. Overlay heat map of the 228 characteristic peak profiles of floral specialized metabolites in Brassicaceae genotypes.

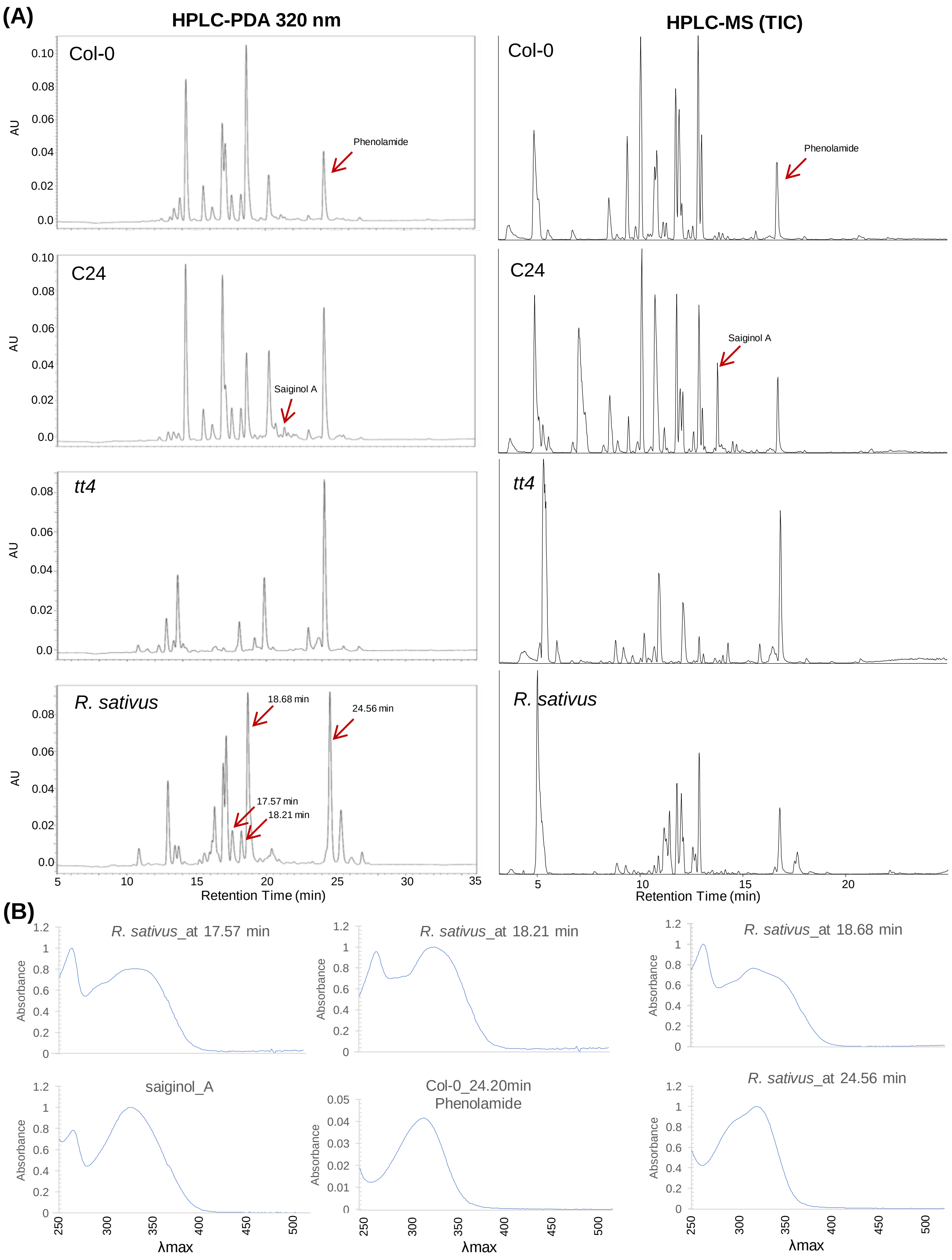

Figure S2. HPLC-PDA and LC-MS Chromatogram of flowers from Arabidopsis accession Col-0, C24, *tt4* mutant as well as *R. sativus* and typical absorption spectrum of phenylaceylated compounds.
(A). HPLC chromatogram detected at 320 nm. Chromatographic peaks at 17.57 min, 18.21 min,18.68 min and 24.56 min of *R. sativus* were indicated by red arrows. (B). Absorption spectrum of peak at 17.57, 18.21, 18.68, 24.56 min from *R. sativus*, saiginol from C24 accession of Arabidopsis (Tohge et al., 2016) and phenolamide from Col-0 were shown. Abbr: AU indicates absorbance unit.
